# Baseline Prevalence of Oral Human Papillomavirus in Mother-Child Pairs With and Without HIV Infection

**DOI:** 10.1001/jamanetworkopen.2024.51512

**Published:** 2024-12-17

**Authors:** Modupe O. Coker, Nicolas F. Schlecht, Esosa Osagie, Paul Akhigbe, Ozoemene N. Obuekwe, Yana Bromberg, Nosayaba Osazuwa-Peters

**Affiliations:** 1 Department of Oral Biology, School of Dental Medicine, Rutgers University, Newark, New Jersey; 2 Department of Epidemiology, School of Public Health, Rutgers University, Newark, New Jersey; 3 Department of Epidemiology, Geisel School of Medicine at Dartmouth, Hanover, New Hampshire; 4 Department of Cancer Prevention and Control, Roswell Park Comprehensive Cancer Center, Buffalo, New York; 5 Institute of Human Virology Nigeria, Abuja, Nigeria; 6 Department of Oral and Maxillofacial Surgery, School of Dentistry, University of Benin Teaching Hospital, Benin City, Nigeria; 7 Department of Biology, Emory College of Arts and Sciences, Atlanta, Georgia; 8 Department of Head and Neck Surgery & Communication Sciences, Duke University School of Medicine, Durham, North Carolina; 9 Department of Population Health Sciences, Duke University School of Medicine, Durham, North Carolina; 10 Duke Cancer Institute, Duke University, Durham, North Carolina

## Abstract

This cohort study measures the prevalence of oral human papillomavirus (HPV) in women and their children in sub-Saharan African by HIV status.

## Introduction

People with HIV have increased risk of persistent oral human papillomavirus (HPV) infection and associated cancers, including oropharyngeal cancer.^1^ Sub-Saharan Africa has among the highest burden of HIV, with a growing population of children perinatally exposed to and/or infected with HIV.^2^ Little is known about prevalence and concordance of oral HPV subtypes in women with HIV and their children in this region. This cohort study evaluated oral HPV prevalence in mother-child dyads by HIV history.

## Methods

This study is part of a larger cohort of mother-child pairs from the University of Benin Teaching Hospital, Nigeria.^3^ Following ethical approval from Rutgers State University of New Jersey and the University of Benin Teaching Hospital, mothers and their children provided written informed consent and assent, respectively, and were enrolled between May and November 2019. Children were split into 3 groups: perinatally infected with HIV and receiving antiretroviral treatment (infection group), exposed to HIV but not infected (exposed group), and age- and sex-matched children without HIV exposure or infection. The study reporting followed the STROBE guidelines for cohort studies.

Participants provided oral rinse specimens for HPV DNA detection and genotyping for 25 HPV subtypes, including high-risk and low-risk types by the GP5+/6+ PCR-EIA system with LMNX genotyping (Diassay). Prevalence and distribution of oral HPV subtypes in mother-child pairs were compared across study groups using χ^2^ or Fisher exact tests, and mother-child concordance was assessed by the κ statistic. All analyses were conducted in R version 4.4.1 (R Project for Statistical Computing).

## Results

The 96 participants had median (IQR) ages of 44 (41-48) years for mothers and 10 (10-11) years for children (Table). Overall, women with HIV had a higher prevalence of oral HPV, regardless of subtype, compared with women without HIV (prevalence of high-risk HPV: 17% [95% CI, 8%-32%] vs 8% [95% CI, 1%-35%]; low-risk HPV: 31% [95% CI, 18%-47%] vs 25% [95% CI, 9%-53%]) (Figure, A). Among children, those in the exposed group had higher prevalence of high-risk HPV than those in the infection group (33% [95% CI, 14%-61%] vs 21% [95% CI, 9%-40%]) and those in the matched group (8% [95% CI, 1%-35%]) (Figure, B), while children in the infection group had greater prevalence of low-risk HPV types. In children with HIV, HPV-81 was the most prevalent subtype, while in children in the exposed group, HPV-16 had the highest prevalence, followed by HPV-81, HPV-45, and HPV-33. Within-dyad comparisons yielded a κ of 0.7, implying strong agreement across mother-child pairs.

**Table.** Sample Characteristics

Characteristic	Participants by child’s HIV status, No. (%)		
	Infected (n = 24)	Exposed, not infected (n = 12)	Not exposed or infected (n = 12)
**Children**			
Sex			
Male	9 (38)	6 (50)	6 (50)
Female	15 (62)	6 (50)	6 (50)
Age, median (IQR), y	10 (10-11)	10 (10-11)	10 (10-11)
CD4 lymphocyte count, mean (SD), cells/mm^3^	748 (500)	NA	NA
Detectable viral load	13 (54)	NA	NA
**Mothers**			
Age at pregnancy, mean (SD), y	30 (1)	32 (1)	31 (1)
Age at study enrollment, mean (SD), y	41 (1)	42 (1)	41 (1)
Married and monogamous	20 (83)	11 (92)	12 (100)
Heard of HPV vaccination	3 (13)	2 (17)	3 (25)
Duration on antiretroviral treatment, median (IQR), mo	59 (0-103)	56 (0-107)	NA

Abbreviation: NA, not applicable.

**Figure.**
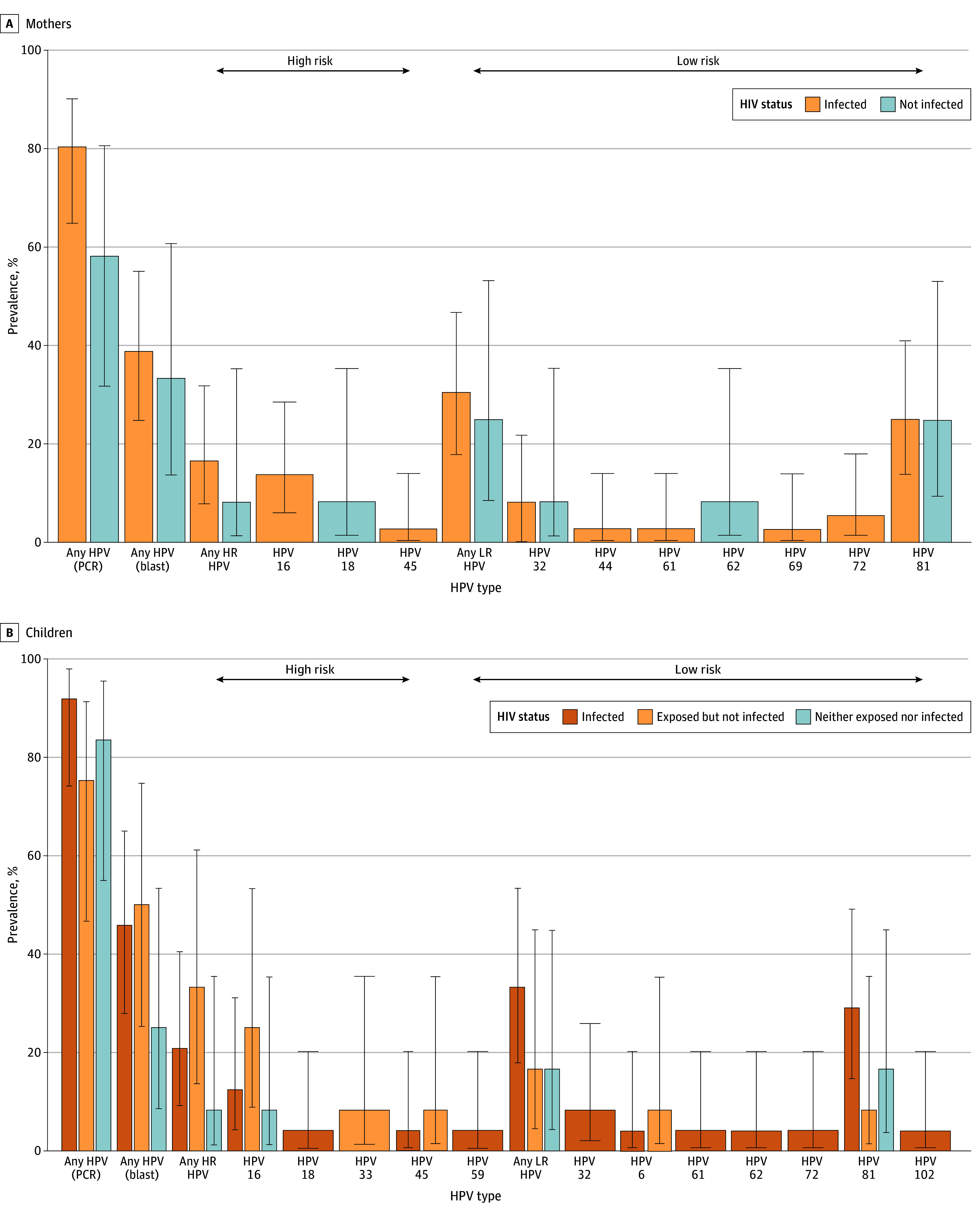
Oral Human Papillomavirus (HPV) Subtype Distribution by HIV Infection Status in All Participants, Based on HIV Exposure Status A, HPV subtype distribution in mothers. B, HPV subtype distribution in children.

## Discussion

We reported baseline prevalence of oral HPV in mother-child pairs in Nigeria, and our data suggest high prevalence of a wide range of oral high- and low-risk HPV subtypes. In particular, low-risk type HPV81 was the most prevalent subtype in all groups except for children exposed to but not infected with HIV. Current literature suggests lower oral HPV prevalence at adolescence, with first peak of infection between age 20 and 25 years.^4^ However, more work is needed to investigate possible differences in oral HPV prevalence among people living with HIV in sub-Saharan Africa. Several US-based epidemiologic studies show higher prevalence of cervical high-risk HPV DNA in adults living with HIV compared with those without infection. Other studies indicate that HPV81 is one of more common cervical types in adult women with HIV; however, the clinical implications are unclear.^5^ If confirmed by larger studies, the high prevalence of HPV81 might have important implications for HPV vaccination, as this subtype is not covered in the nonvalent vaccine currently available.^6^ Additionally, the high prevalence of oral HPV subtypes in women and their children with HIV suggests postnatal transmission and potential role of immune dysregulation in oral HPV carriage, increasing risk of oral HPV persistence and ultimately HPV-associated cancer.

This study was limited by its observational design and lack of power to detect statistically significant differences. Nevertheless, findings highlight the need for larger longitudinal studies in high-risk populations to elucidate factors associated with oral HPV infection and persistence. This is particularly important in sub-Saharan Africa, where HPV vaccine uptake is suboptimal.

## Notes

Supplement.
Data Sharing Statement
