# Supplementary material for: Baseline Prevalence of Oral Human Papillomavirus in Mother-Child Pairs With and Without HIV Infection
Source: JAMA Netw Open. 2024 Dec 17;7(12):e2451512. doi: 10.1001/jamanetworkopen.2024.51512 (PMC11653123; doi:10.1001/jamanetworkopen.2024.51512)
Supplement: Supplement. — Data Sharing Statement [file jamanetwopen-e2451512-s001.pdf]

Data Sharing Statement

Coker. Baseline Prevalence of Oral Human Papillomavirus in Mother-Child Pairs With and Without HIV Infection. *JAMA Netw Open*. Published December 17, 2024. doi:10.1001/jamanetworkopen.2024.51512

Data
Data available: No

Additional Information
Explanation for why data not available: Data is sensitive in this vulnerable population, and there are concerns about identification given the small sample size.
